# Assessment of regional right ventricular systolic function in patients with obstructive sleep apnea syndrome using velocity vector imaging: Erratum

**DOI:** 10.1097/MD.0000000000006211

**Published:** 2017-02-17

In the article “Assessment of regional right ventricular systolic function in patients with obstructive sleep apnea syndrome using velocity vector imaging”,^[1]^ which appeared in Volume 95, Issue 35 of *Medicine*, Zhibin Wang's name was originally spelled incorrectly in the byline.
